# Beneficial Effects of *Limosilactobacillus fermentum* CECT 5716 Administration to Infants Delivered by Cesarean Section

**DOI:** 10.3389/fped.2022.906924

**Published:** 2022-07-07

**Authors:** Ruth Blanco-Rojo, José Maldonado, Monika Schaubeck, Metehan Özen, Eduardo López-Huertas, Mónica Olivares

**Affiliations:** ^1^Research and Development Department, Biosearch Life, a Kerry Company, Granada, Spain; ^2^Pediatric Unit, University Hospital Virgen de las Nieves, Granada, Spain; ^3^Department of Pediatric, University of Granada, Granada, Spain; ^4^Biosanitary Research Institute (IBS), Granada, Spain; ^5^HiPP GmbH & Co. Vertrieb KG, Pfaffenhofen, Germany; ^6^Department of Pediatrics, School of Medicine, Acibadem University, Istanbul, Turkey; ^7^Estación Experimental Zaidín, Consejo Superior Investigaciones Científicas, Granada, Spain

**Keywords:** cesarean section, probiotics, infant formula, gastrointestinal infections, respiratory infections

## Abstract

Cesarean section (CS) disrupts the natural microbiota colonization process in infants, which might compromise immune system maturation, leading to a higher risk of infections. We evaluated the effect of the probiotic *Limosilactobacillus (L.) fermentum* CECT 5716 on the incidence of gastrointestinal and respiratory infections in the CS infant subgroups (*n* = 173) of three randomized clinical trials in which this probiotic strain was demonstrated to be safe and effective for preventing infections. Therefore, the data for the CS infants were extracted to obtain the incidence rate ratio (IRR) and 95% CI for gastrointestinal and respiratory infections for each study and were then combined to obtain a pooled IRR and 95% CI using the generic inverse variance method. There was a significant reduction of 73% in the incidence of gastrointestinal infections in CS infants receiving *L. fermentum* CECT 5716 compared with those receiving the control formula [*n* = 173, IRR: 0.27 (0.13, 0.53), *p* = 0.0002]. Regarding respiratory infections, although pooled results showed a reduction of 14% in the probiotic group, the difference was not statistically significant [*n* = 173, IRR (95% CI): 0.86 (0.67, 1.11), *p* = 0.25]. In conclusion, the administration of *L. fermentum* CECT 5716 to CS-born infants protects them from gastrointestinal infections by reducing the risk by up to 73% in this population.

## Introduction

Vaginal delivery (VD) allows contact of the neonate with the vaginal and enteric microbiota of the mother and therefore influences infants' gut colonization. CS, apart from disrupting this natural colonization process, also imply exposition of the newborn to microbes from the operating theater and perinatal antibiotics, thus promoting a significantly different microbiota compared to that in VD infants (1). Specifically, the microbiota of VD infants is characterized by microbes including *Limosilactobacillus, Prevotella, Bacteroides, Escherichia/Shigella, Bifidobacterium* spp. and other members of the former genus *Lactobacillus* (2). In contrast, *Staphylococcus, Streptococcus, Corynebacterium, Veillonella* and *Propionibacterium* spp. dominate in the microbiome of CS delivered infants (3, 4) and delay the expected normal intestinal colonization. Moreover, lower amounts of total intestinal bacteria and lower diversity have been observed in CS infants (5–7).

Immune system maturation is dependent on intestinal colonization. The gut-associated lymphoid tissues, such as the Peyer's patches, the mesenteric lymph nodes and the isolated lymphoid follicles require signals from the intestinal microbiota to ensure complete development and maturation. This learning process determines an individual's immune response throughout their life (8–10). The close relationship between the colonization process in infants and the development of the immune system explains, at least in part, the immunological differences in CS children compared to VD children (6, 9). These changes in the immune system due to CS microbiota dysbiosis have been related to a higher risk of infections and an increased frequency of hospital admissions due to any type of infection in CS infants (11–13).

To reduce the potential negative effects of CS delivery on early colonization, strategies to imitate natural microbial colonization by VD have been evaluated, including exposure of infants to the mother's fecal and/or vaginal microbiota (14, 15). Although these strategies may resemble the natural microbiome colonization process during delivery, the physician performed, artificial application is under intense debate due to the high risk of pathogen transmission to infants (16–18). It is important to highlight that breastfeeding seems to help to counteract the deleterious effect of CS on the microbiota without safety concerns related to fecal or vaginal transplant procedures and, therefore, breastfeeding should be encouraged and support should be given by physicians and midwifes (19). However, women who deliver babies by CS are less likely to breastfeed or delay breastfeeding initiation (20). Therefore, if breastfeeding is not possible or insufficient, a feasible strategy would be to introduce bacteria naturally present in human milk into infant formulae.

*Limosilactobacillus (L.) fermentum* CECT 5716, previously named *Lactobacillus fermentum* CECT 5716 (2), is a probiotic strain originally isolated from human milk (21). Three randomized clinical trials (RCTs) (22–24) performed in infants demonstrated the safety of the probiotic strain as well as its usefulness for preventing community-acquired infections, such as gastrointestinal and respiratory infections (Table 1).

**Table 1:** Summary of the incidence of gastrointestinal and respiratory infections in infants.

	**N**°**events**	**Incidence rate (SD)**	**N**°**events**	**Incidence rate (SD)**	**Incidence rate ratio (95% CI)**	***P*-value**
**Maldonado et al**. **(****22****)** **(n** **=** **188)** **Time of intervention 6–12 months**	**Control group (*****n** **=*** **91)**		**Experimental group (*****n** **=*** **97)**			
Gastrointestinal infections	33	0.363 (0.53)	19	0.196 (0.51)	0.54 (0.31–0.95)	0.032
Respiratory infections	134	1.470 (1.31)	106	1.093 (1.00)	0.74 (0.58–0.96)	0.022
**Gil-Campos et al**. **(****23****)** **(n** **=** **121)** **Time of intervention 1–6 months**	**Control group (*****n** **=*** **60)**		**Experimental group (*****n** **=*** **61)**			
Gastrointestinal infections	17	0.283 (0.07)	5	0.082 (0.04)	0.289 (0.085–0.831)	0.018
Respiratory infections	43	0.716 (0.11)	42	0.689 (0.11)	0.977 (0.623–1.530)	0.933
**Maldonado et al**. **(****24****)** **(n** **=** **126)** **Time of intervention 1–12 months**	**Control group (*****n** **=*** **61)**		**Experimental group (*****n** **=*** **65)**			
Gastrointestinal infections	42	0.689 (0.106)	25	0.385 (0.077)	0.559 (0.326–0.938)	0.014
Respiratory infections*	162	2.98 (0.235)	217	3.45 (0.234)	1.155 (0.941–01.417)	0.324

*Data was collected for the published studies (22–24). Respiratory infections included the sum of upper and lower tract respiratory infections.*

*Unpublished data, analyzed by Poisson regression model adjusted by sex, age at entry and breastfeeding before the intervention.

These three studies involved both VD- and CS-born infants. The objective of the present study is to analyze the effect of *L. fermentum* CECT 5716 consumption on the incidence of gastrointestinal and respiratory infections in the CS subgroup. Therefore, data on CS-born infants in the three aforementioned clinical trials were extracted separately, and the obtained results were pooled in a meta-analysis.

## Materials and Methods

The protocols for the three randomized clinical trials have been previously discussed in detail (22–24). Briefly, all the studies were double-blind, randomized, controlled trials and included healthy infants exclusively fed formula, with an inclusion age ranging from 1 month (23, 24) to 6 months (22) (Table 1). In all the studies, *Limosilactobacillus fermentum* CECT 5716 (HEREDITUM^®^ LC40) was administered as an ingredient in a powdered infant formula with a nutritional composition in accordance with current EU regulations. All formulae were well tolerated, and compliance was good. Furthermore, no adverse effects related to formula consumption were reported. The CS rates in the studies were 31% in Maldonado et al. (24), 43% in Gil-Campos et al. (23) and 44% in Maldonado et al. (22).

For the purpose of this study, only data for CS-born infants were taken into consideration. The main outcomes of the current study were the incidence of gastrointestinal infections and respiratory infections. In all the studies, the diagnosis of infections was performed by pediatricians based on specific symptoms and standardized definitions, as followed. Gastrointestinal infection was defined as loose or watery stools ≥3 times/day, with or without fever or vomiting in Maldonado et al. (24), and in Gil-Campos et al. (23); whereas in Maldonado et al. (22) was defined as the occurrence of loose or watery stool ≥4 times/day, with or without a fever or vomiting. Respiratory tract infections were defined as the presence of abundant mucosity and/or cough during ≥2 consecutive days with or without fever or the presence of wheezing and/or crepitants with or without fever in all three studies (22–24).

The occurrence of gastrointestinal and respiratory infections was described for each study using the incidence rate (IR) and the incidence rate ratio (IRR) with the 95% CI and *p-*value for the IRR in each clinical trial. A Poisson regression model was applied to adjust the number of events by sex, age at intervention entry, and whether infants were breastfed before the intervention.

The IRR and 95% CI for gastrointestinal and respiratory infections were extracted from individual studies and combined to obtain a pooled IRR and 95% CI by combining the effect-size estimates (b-coefficients and 95% CIs) from the 3 studies, which were weighted by the inverse of the corresponding standard errors (SEs).

A general alpha level of 0.05 was used as the cutoff point for statistical significance. Statistical analyses were carried out using SPSS software version 27.0 for Windows (SPSS, Chicago, IL, USA) and RevMan 5.4 (25).

## Results

**Figure 1:**
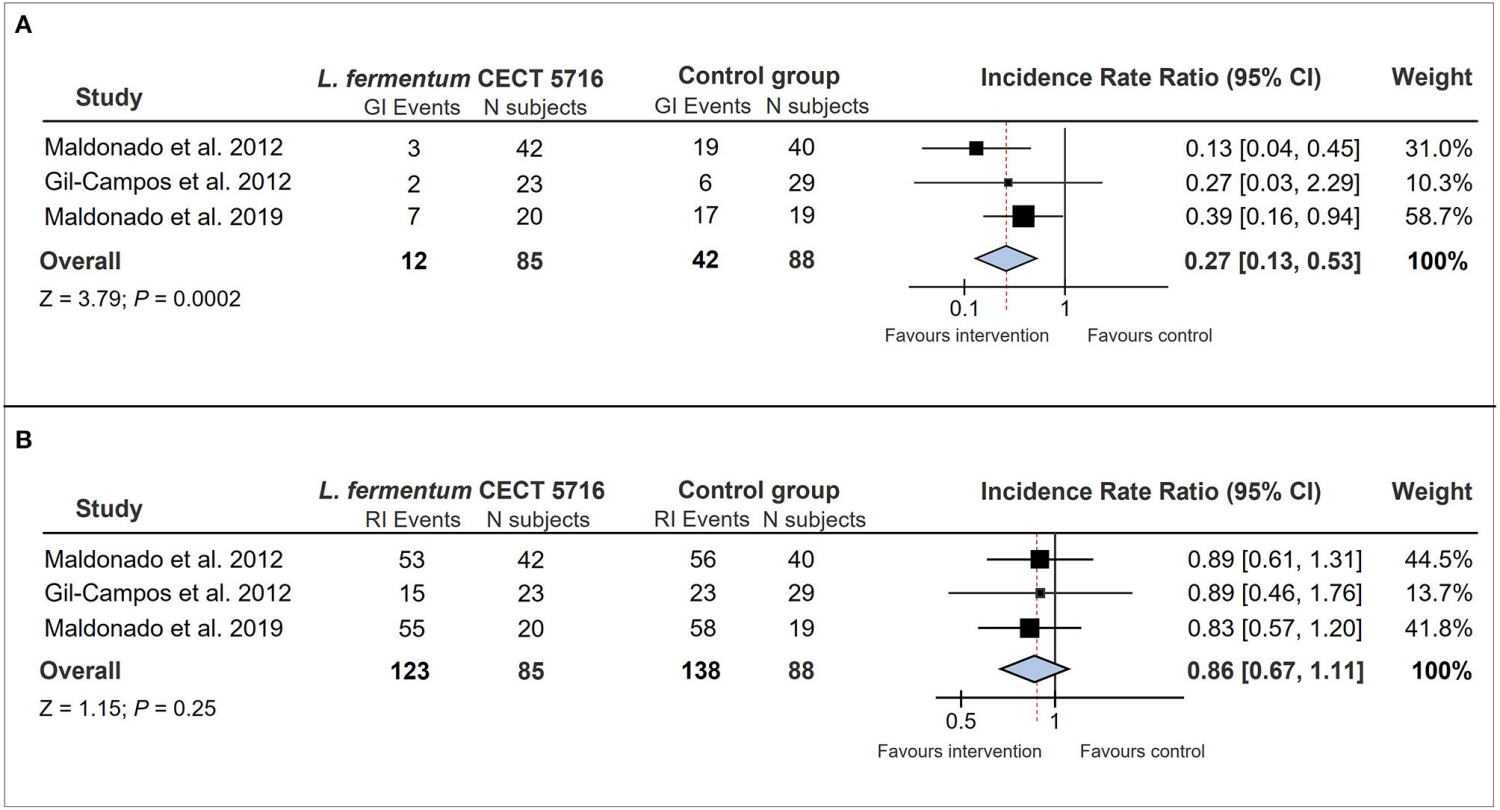
Forest plots illustrating the effect of *L. fermentum* CECT 5716 interventions on **(A)** the incidence of gastrointestinal infections and **(B)** the incidence of respiratory infections in infants born by CS. CI, confidence interval; GI, gastrointestinal infection; RI, respiratory infection; N subjects, number of volunteers participating in each study-intervention group.

Analysis of data from CS infants showed that the incidence of gastrointestinal infections was reduced by the consumption of *L. fermentum* CECT 5716 (Table 2), reaching significance in two of the three RCTs analyzed separately. Furthermore, pooled results from the 173 CS infants showed a significant reduction of 73% in the incidence of gastrointestinal infections in CS-born children receiving *L. fermentum* CECT 5716 in comparison to infants receiving the control formula [*n* = 173, IRR: 0.27 (0.13, 0.53), *p* = 0.0002] (Figure 1A).

**Table 2:** Incidence of gastrointestinal and respiratory infections in infant born by CS.

	**N**°**events**	**Incidence rate (SD)**	**N**°**events**	**Incidence rate (SD)**	**Incidence rate ratio (95% CI)**	*P*-value*
**Maldonado et al**. **(****22****)** **(n** **=** **82)** **Time of intervention 6–12 months**	**Control group (*****n** **=*** **40)**		**Experimental group (*****n** **=*** **42)**			
Gastrointestinal infections	19	0.48 (0.112)	3	0.06 (0.037)	0.132 (0.039–0.449)	0.001
Respiratory infections	56	1.40 (0.190)	53	1.25 (0.175)	0.894 (0.609–1.314)	0.570
**Gil-Campos et al**. **(****23****)** **(n** **=** **52)** **Time of intervention 1–6 months**	**Control group (*****n** **=*** **29)**		**Experimental group (*****n** **=*** **23)**			
Gastrointestinal infections	6	0.18 (0.043)	2	0.04 (0.043)	0.273 (0.032–2.290)	0.231
Respiratory infections	23	0.74 (0.162)	15	0.67 (0.162)	0.897 (0.458–1.756)	0.751
**Maldonado et al**. **(****24****)** **(n** **=** **39)** **Time of intervention 1–12 months**	**Control group (*****n** **=*** **19)**		**Experimental group (*****n** **=*** **20)**			
Gastrointestinal infections	17	0.89 (0.219)	7	0.35 (0.132)	0.387 (0.158–0.943)	0.037
Respiratory infections	58	3.23 (0.426)	55	2.67 (0.367)	0.828 (0.570–1.202)	0.320

*Poisson regression model adjusted by sex, age at entry and breastfeeding before the intervention.

Analysis of the incidence of respiratory infections (RI) showed no significant reduction by *L. fermentum* CECT 5716 in CS-delivered infants (Table 2), although a decreasing trend of these types of infections was observed. The pooled results showed a 14% reduction in the incidence of respiratory infections in CS-born infants receiving the probiotic, although the difference was not statistically significant [*n* = 173, IRR: 0.86 (0.67, 1.11), *p* = 0.25] (Figure 1B).

## Discussion

The present study shows that the human milk-isolated probiotic strain *L. fermentum* CECT 5716 seems to be effective in reducing the incidence of gastrointestinal infections in CS-born and formula fed infants. A recent meta-analysis, which included the total population of the three clinical trials (*n* = 512), showed a significant overall reduction in the incidence of gastrointestinal infections of 46% (*p* = 0.0004) (26). However, the effect observed in the specific population born by CS shows an even higher level of reduction (73%), suggesting that the protective effect of this probiotic strain might be even more relevant in the case of CS delivery.

Infectious diseases are the most common type of illness for infants worldwide, resulting in the leading cause of morbidity and mortality during the first year of life (22, 27). Among the risk factors for infections, increasing evidence showed a higher risk for gastrointestinal and respiratory infections, and related hospitalizations in CS infants (12, 28–30). Different studies have been performed to evaluate the effect of probiotic administration to infants born by CS (31–34). However, most of these studies have focused primarily on evaluating the impact on the microbiota. Consequently, there are still few data on whether probiotics may help in the maturation of the immune system and thereby reduce the risk of infections in infants with CS. Therefore, the results of the present study add new evidence regarding the usefulness of administering a probiotic strain in the prevention of community-acquired infections in CS infants, which could be of great interest in this population.

It is interesting to note that the incidence rates of both gastrointestinal and respiratory infections were lower in the study of Gil-Campos et al. (23). Here, infants were followed-up from 1 to 6 months, compared to the other two studies in which the infants were in the study from 6 to 12 months (22) and from 1 to 12 months of age (24). This observation is in agreement with other studies (35), and could be explained by several factors, including the fact that infants that may attend day care centers in Spain—a major risk factor for infection in infants (36–38)—are normally older than 6 months of age (38).

Different mechanisms influenced by gut colonization in early life are involved in protection against gastrointestinal infections, e.g., competition with pathogenic bacteria, production of bacteriocins, strengthening of the epithelial barrier and therefore reduction of intestinal permeability, or modulation of the host's local immune response by inducing the production of antimicrobial proteins (39, 40). Therefore, these mechanisms could be affected in the case of CS-associated dysbiosis. *L. fermentum* CECT 5716 has been shown to support pathogen defense through all these mechanisms in different *in vitro* and *in vivo* models (41). Interestingly, results obtained in newborn rats show a protection in intestinal barrier integrity during stress induced by maternal separation (42). The fact that *L. fermentum* CECT 5716 can enhance the immune response (43, 44) as it interferes with the mechanisms involved in enteropathogen internalization (45) might be responsible for the improved protective effect observed against gastrointestinal infections. Interestingly, in adults receiving anti influenza vaccination, the consumption of *L. fermentum* CECT 5716 induced an increase in specific IgA (44). The immunoglobulin A (IgA) is the predominant antibody isotype in the mucosal immune system, which widely exists in the gastrointestinal tract, respiratory tract, vaginal tract, tears, saliva, and colostrum (46). It acts as the first line of defense against pathogens. There is a lack of IgA-secreting B cells in neonates until exposure to bacteria, suggesting that the commensal microorganisms were able to induce sIgA secretion (47). Activation of IgA production might be related to *L. fermentum* CECT 5716 capability to interact with other immune cells inducing immune response modulating cytokine release (48). Moreover, consumption of *L. fermentum* CECT 5716 has been shown to increase the load of fecal lactobacilli and bifidobacteria (22, 24). Therefore, the protective effect of this probiotic strain on gastrointestinal infections might also be due to its ability to modulate the microbiota (3, 6, 7). However, as the bacterial quantification methods were different in the three clinical trials included in this analysis, no evaluation of the overall effect of the probiotic strain on the microbiota of the CS-born infants could be made. A more complete analysis of the microbiota in infants and studies focusing on CS-born infants should be performed in the future to elucidate the role of probiotic intervention on the microbiota and its impact on the immune response and susceptibility to infections.

The effect of *L. fermentum* CECT 5716 on respiratory infections has been attributed to the modulation of the immune response (22, 44). However, the effect in the CS-born population was not as obvious for respiratory infections. In the analysis of the total population in the study reported by Maldonado et al. (22), a reduction in the incidence of respiratory infections in the group consuming the probiotic strain was observed (IRR = 0.74; CI 95% 0.58–0.96; *p* = 0.022). Separate analyses of upper and lower respiratory infections showed that the difference was due to the effect on upper respiratory infections (19). In the second study of Maldonado et al. (24), a significant reduction in upper respiratory infections in the CS-born subgroup (IRR = 0.492; CI 95% 0.294–0.815; *p*= 0.006) was observed. In contrast, the analysis of the CS-born subpopulation performed in the present study considering total respiratory infections (upper and lower respiratory infections) did not show a significant effect of the probiotic intervention. As Gil-Campos et al. (23) did not distinguish between upper and lower infections, a global analysis of upper infections could not be performed in the present analysis of the CS-born infant subpopulation. Moreover, no blood samples were collected in any of these studies, so the effect of the probiotic intervention on the immune response in these children could not be analyzed. In addition, the duration and/or severity of the respiratory infections were not recorded. Therefore, further studies to elucidate the effect of *L. fermentum* CECT 5716 on the prevention and on the severity and duration of respiratory infections in CS infants should be performed.

Some limitations of the study should be noted. First, we included data from three different studies that followed-up the infants in different age ranges, from 1 to 6 months (23), 6 to 12 months (22), and 1 to 12 months old (24). However, we pooled the data by using the most adequate methodology (49). Second limitation is that one of the studies (22) used a different dose of the probiotic. However, results obtained in a previous meta-analysis did not support a dose–response effect for *L. fermentum* CECT 5716 (26). This observation is also in agreement with the non-dependent dose effect exerted by *L. fermentum* for other applications (50). Another limitation is that the recorded data did not allow to distinguish between scheduled and emergency CS (51). Finally, although infectious diseases were diagnosed by a pediatrician, definition for gastrointestinal infections were slightly differed in one of the three studies (22).

In conclusion, the administration of *L. fermentum* CECT 5716 to CS-born infants protects them from gastrointestinal infections by reducing the risk of this type of infection by up to 73% in this population. The protective effect of this probiotic has been extensively demonstrated in several clinical trials, but the results of the present study suggest that it could be even more relevant in the case of CS-born infants, a population at higher risk for infections. The use of probiotics in this specific population should be more deeply studied as a strategy to balance the negative effects of CS on immune system maturation.

## Data Availability Statement

The data analyzed in this study is subject to the following licenses/restrictions: Due to the nature of this research, participants of this study did not agree for their data to be shared publicly, so supporting data is not available. Requests to access these datasets should be directed to rblanco@biosearchlife.com.

## Ethics Statement

The studies involving human participants were reviewed and approved by Regional Ethics Committee of the Sistema Andaluz de Salud, Sevilla, Spain. Written informed consent to participate in this study was provided by the participants' legal guardian/next of kin.

## Author Contributions

RB-R designed the methodology, analyzed the data, and contributed to the manuscript writing. MS and MÖz contributed to the manuscript writing and critically revised the manuscript. JM critically revised the manuscript. EL-H participated in the conception of the study and critically revised the manuscript. MOl designed the study, interpreted the results, and wrote the draft of the manuscript. All authors have read and approved the final manuscript.

## Conflict of Interest

RB-R and MOl are employees of Biosearch Life, a Kerry Company. Biosearch Life, a Kerry Company, is the owner of the patent for *L. fermentum* CECT5716. MS is an employee at HiPP. The remaining authors declare that the research was conducted in the absence of any commercial or financial relationships that could be construed as a potential conflict of interest.

## Publisher's Note

All claims expressed in this article are solely those of the authors and do not necessarily represent those of their affiliated organizations, or those of the publisher, the editors and the reviewers. Any product that may be evaluated in this article, or claim that may be made by its manufacturer, is not guaranteed or endorsed by the publisher.
